# Circulating Malondialdehyde Concentrations in Obstructive Sleep Apnea (OSA): A Systematic Review and Meta-Analysis with Meta-Regression

**DOI:** 10.3390/antiox10071053

**Published:** 2021-06-29

**Authors:** Maria Carmina Pau, Elisabetta Zinellu, Sara S. Fois, Barbara Piras, Gianfranco Pintus, Ciriaco Carru, Arduino A. Mangoni, Alessandro G. Fois, Angelo Zinellu, Pietro Pirina

**Affiliations:** 1 Department of Medical, Surgical and Experimental Sciences, University of Sassari, 07100 Sassari, Italy; mcpau@uniss.it (M.C.P.); s.fois40@studenti.uniss.it (S.S.F.); barbara.piras@aousassari.it (B.P.); agfois@uniss.it (A.G.F.); 2 Clinical and Interventional Pneumology, University Hospital Sassari (AOU), 07100 Sassari, Italy; elisabetta.zinellu@aousassari.it; 3 Department of Biomedical Sciences, University of Sassari, 07100 Sassari, Italy; gpintus@uniss.it (G.P.); carru@uniss.it (C.C.); azinellu@uniss.it (A.Z.); 4 Department of Medical Laboratory Sciences, Sharjah Institute for Medical Research, College of Health Sciences, University of Sharjah, 27272 Sharjah, United Arab Emirates; 5 College of Medicine and Public Health, Flinders University, Bedford Park, SA 5042, Australia; arduino.mangoni@flinders.edu.au; 6 Department of Clinical Pharmacology, Flinders Medical Centre, Bedford Park, SA 5042, Australia

**Keywords:** obstructive sleep apnea, lipid peroxidation, malondialdehyde, oxidative stress

## Abstract

Oxidative stress induced by nocturnal intermittent hypoxia plays a significant pathophysiological role in obstructive sleep apnea (OSA). Malondialdehyde (MDA), one of the most commonly investigated markers of lipid peroxidation, might assist with the monitoring of oxidative balance in OSA. We conducted a systematic review and meta-analysis to evaluate the differences in circulating MDA concentrations between patients with OSA and non-OSA controls. A systematic search was conducted in the electronic databases Pubmed, Web of Science, Scopus and Google Scholar from inception to December 2020 by using the following terms: “malondialdehyde” or “MDA”; and “Obstructive Sleep Apnea Syndrome”, “OSAS” or “OSA”. We identified 26 studies in 1223 OSA patients and 716 controls. The pooled MDA concentrations were significantly higher in patients with OSA (standardized mean difference (SMD) 1.43 μmol/L, 95% confidence interval (CI) 1.03 to 1.83 μmol/L, *p* < 0.001). There was extreme heterogeneity between the studies (I^2^ = 92.3%, *p* < 0.001). In meta-regression analysis, the SMD was significantly associated with age, the assay type used and publication year. In our meta-analysis, MDA concentrations were significantly higher in OSA patients than in controls. This finding suggests that MDA, which is a marker of lipid peroxidation, is involved in the pathogenesis of OSA and provides insights for future studies investigating its potential clinical use.

## 1. Introduction

The obstructive sleep apnea syndrome (OSA) is a common breathing-related sleep disorder that affects over 900 million people worldwide and results in impaired quality of life and increased risk of motor vehicle accidents and cardiovascular diseases [1,2]. OSA is characterized by intermittent and repeated episodes of collapse of the upper airway during sleep, resulting in partial (hypopnea) or complete (apnea) airflow obstruction with consequent hypoxia and reoxygenation [3]. The fluctuations in oxygen saturation resemble the phenomenon of ischemia-reperfusion injury, which causes mitochondrial dysfunction and stimulates the production of reactive oxygen species (ROS) [4,5,6]. The excessive ROS generation results in oxidation and consequent structural and functional damage of proteins, DNA and lipids [7]. In OSA patients, oxidative stress appears to be a major contributor to the adverse outcomes associated with this syndrome, particularly cardiovascular morbidity, vascular damage and endothelial dysfunction [8,9]. Lipid peroxidation, which is a direct consequence of oxidative stress, causes further oxidative damage in membranes, lipoproteins and other molecules that contain lipids. The varieties of secondary products, e.g., lipid hydroperoxides and various aldehydes, are generated during this process [10]. Malondialdehyde (MDA), one of the major aldehyde species, is produced by the peroxidative decomposition of unsaturated fatty acids [10]. This molecule represents one of the most studied indicators of lipid peroxidation degree and is measured as a biomarker of oxidative stress in different diseases, including chronic obstructive pulmonary disease, idiopathic pulmonary fibrosis and several cancers [11,12,13,14]. Several studies have assessed MDA concentrations in OSA patients and controls, with conflicting results [15,16,17]. Therefore, the biological and clinical role of MDA in OSA is not well established. We sought to address this issue by performing a systematic review and meta-analysis to evaluate the presence and the effect size of the differences in the blood concentrations of MDA between patients with OSA and controls. A meta-regression was also conducted to investigate possible associations between effect size and specific patient, study design and analytical characteristics.

## 2. Methods

### 2.1. Search Strategy, Eligibility Criteria and Study Selection

A systematic search of the literature in the electronic databases such as Pubmed, Web of Science, Scopus and Google Scholar, from inception to December 2020, was performed using the following keywords and their combinations: “malondialdehyde” or “MDA”; and “Obstructive Sleep Apnea Syndrome”, “OSAS” or “OSA”. Two investigators independently performed the literature search by screening the abstracts. If they were found to be relevant, then the full articles were reviewed. The Eligibility criteria included the following: (i) analysis of MDA concentrations in plasma or serum; (ii) comparison of subjects with or without OSA (case-control design); (iii) adult patients; (iv) ≥10 patients with OSA; (v) studies reporting apnea-hypopnea index (AHI) values; (vi) English language; and (vii) full-text available. The references of individual articles were also reviewed to identify additional studies. Any discrepancy between the reviewers was resolved by a third investigator. The quality of each study was assessed using the Newcastle-Ottawa Scale (NOS) [18].

### 2.2. Statistical Analysis

In order to assess the differences in MDA concentrations between OSA and non-OSA subjects, standardized mean differences (SMD) were measured to set up forest plots of continuous data. The test was considered statistically significant when the *P* value was <0.05 and 95% confidence intervals (CIs) were reported. If necessary, the mean and standard deviation were extrapolated from the median and interquartile range, as previously reported by Wan et al. [19], from the median and range, as described by Hozo et al. [20], or from individual graphs using the Graph Data Extractor software. Heterogeneity of SMD across studies was examined using the Q statistic (the significance level was set at *p* < 0.10). The inconsistency between studies was quantitatively measured by I^2^ statistic (I^2^ < 25%—no heterogeneity; I^2^ between 25% and 50—moderate heterogeneity; I^2^ between 50% and 75%—large heterogeneity; I^2^ > 75%—extreme heterogeneity) [21,22]. Statistical heterogeneity was defined as an I^2^ statistic value ≥50% [22]. A random-effects model was used if the heterogeneity was high. Additionally, a sensitivity analysis was performed to test the influence of each study on the overall risk estimate by excluding one study at a time [23]. The presence of publication bias was evaluated by the analysis of the relation between study size and magnitude of effect using the Begg’s adjusted rank correlation test and the Egger’s regression asymmetry test at the *p* < 0.05 level of significance [24,25]. The presence of publication bias was further tested and eventually corrected using the Duval and Tweedie “trim and fill” procedure [26]. Confidence intervals at 95% (CIs) were reported for each effect size and the overall effect and *p* < 0.05 indicated statistical significance. Statistical analyses were performed using Stata 14 (STATA Corp., College Station, TX, USA). The study followed the principles defined in the PRISMA statement for reporting systematic reviews and meta-analyses [27].

## 3. Results

**Figure 1:**
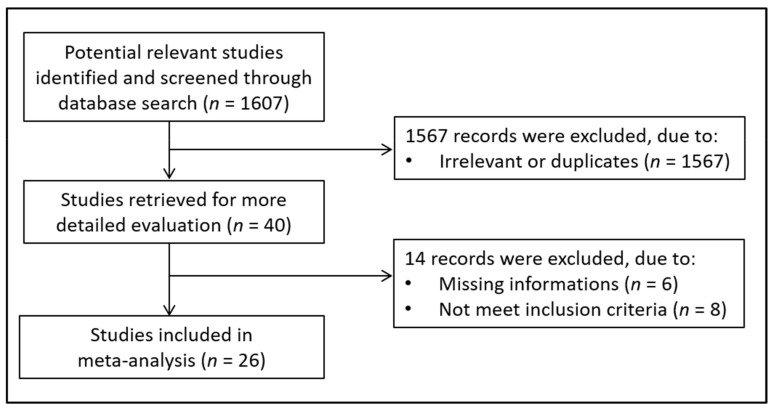
Flow chart of study selection.

A flow chart describing the screening process is presented in Figure 1. The systematic search initially identified 1607 studies. After the first screening, 1567 were excluded because they were either duplicates or irrelevant. After a full-text revision of the remaining 40 articles, 14 were further excluded because of missing information or non-compliance with the inclusion criteria. Thus, 26 studies in 1233 OSA patients (mean age 49 years, 82% males) and 716 controls (mean age 48 years, 76% males) published between 2005 and 2018 were included in the meta-analysis (Table 1) [17,28,29,30,31,32,33,34,35,36,37,38,39,40,41,42,43,44,45,46,47,48,49,50,51,52]. The study by Wang L. et al. 2010 [34] divided OSA patients into two groups according to age (elderly and non- elderly patients). Therefore, this study was analyzed by considering the two groups separately in the following manner: (a) elderly and (b) non-elderly.

In all studies, OSA was diagnosed by polysomnography. Patients were recruited from a sleep center in 20 out of 26 studies. In the remaining six studies, patients were recruited from other clinical cohorts in two of the studies whereas no information regarding the recruitment source was provided in four of the studies.

**Figure 2:**
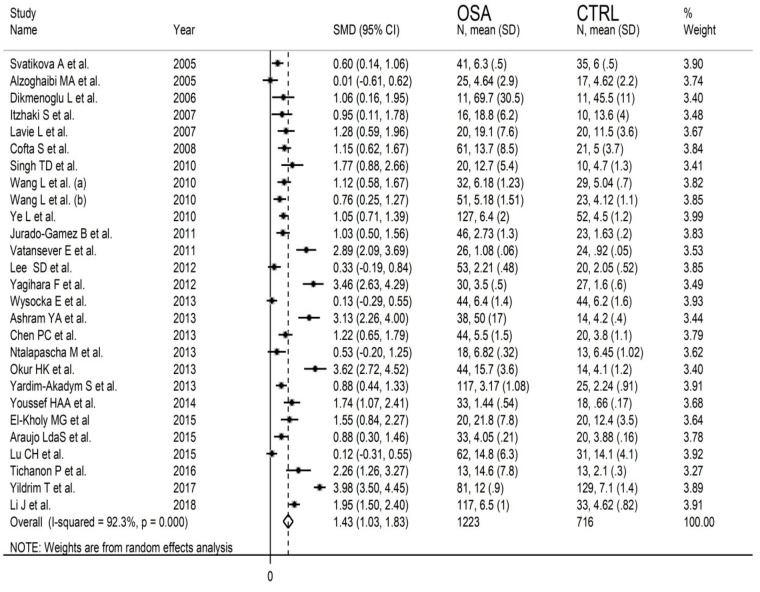
Forest plot of studies investigating MDA concentrations in OSA patients and controls.

**Figure 3:**
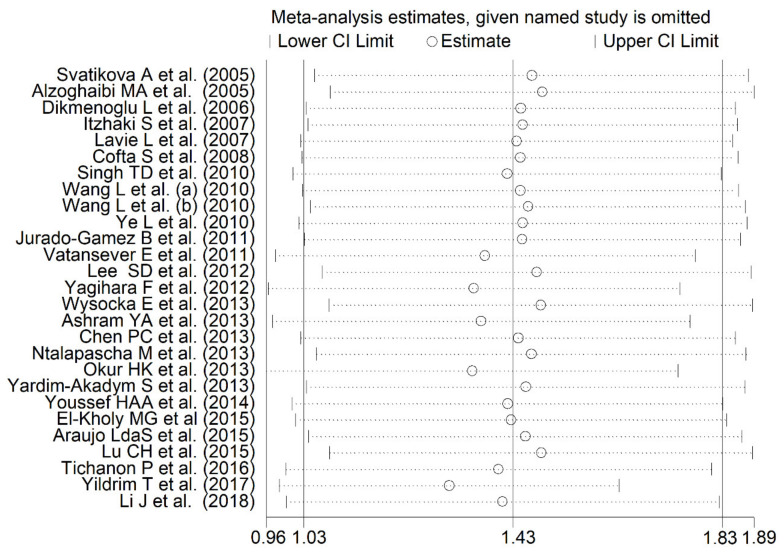
Sensitivity analysis of the association between MDA and OSA. The influence of individual studies on the overall standardized mean difference (SMD) is shown. The middle vertical axis indicates the overall SMD and the two vertical axes indicate the 95% confidence intervals (CIs). The hollow circles represent the pooled SMD when the remaining study is omitted from the meta-analysis. The two ends of each broken line represent the 95% CIs.

**Figure 4:**
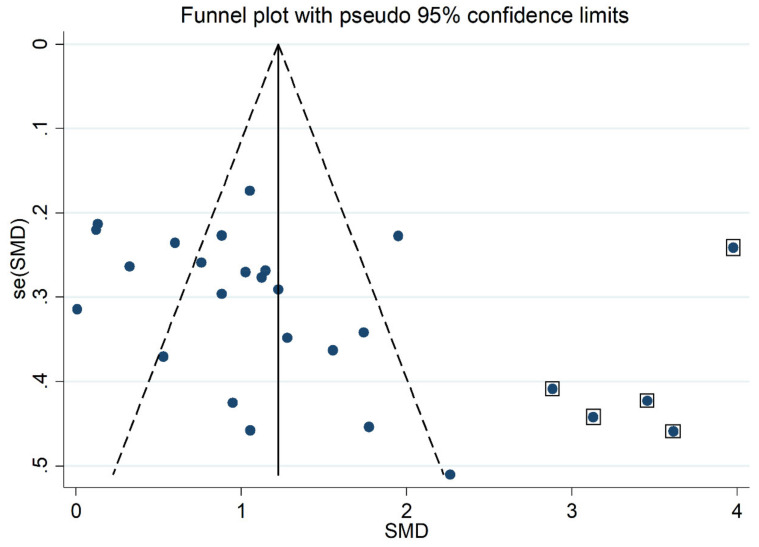
Funnel plot of studies investigating MDA concentrations in OSA. The enclosed circles represent the five studies with a likely distortion effect on the funnel plot symmetry.

**Figure 5:**
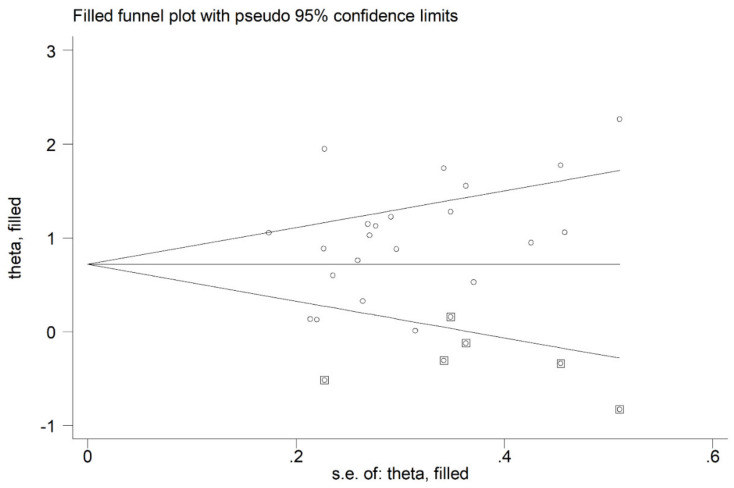
Funnel plot of studies investigating MDA concentrations in OSA after trimming and filling. Dummy studies and genuine studies are represented by enclosed circles and free circles, respectively.

MDA concentrations in the included studies and the forest plot for MDA levels in OSA patients and controls are reported, respectively, in Table 2, Supplementary Figure (Figure S1) and Figure 2. In all studies, OSA patients had higher MDA concentrations compared to the controls (mean difference range, 0.01 to 3.98), although the difference was not statistically significant in five of the studies [28,38,40,43,49]. Our analysis revealed a substantial heterogeneity between studies (I^2^ = 92.3%, *p* < 0.001). Thus, random-effects models were used. Overall, pooled results showed significantly higher MDA levels in patients with OSA (SMD 1.43, 95% CI 1.03 to 1.83, *p* < 0.001). The corresponding pooled SMD values were not altered when each study was consecutively removed as shown by sensitivity analysis (effect size range, between 1.31 and 1.49, Figure 3). The funnel plot (Figure 4) indicated a possible distortive effect of five studies on the right side of the graph [37,39,41,44,51]. Their exclusion attenuated both the effect size (SMD 0.98, 95% CI 0.73 to 1.22, *p* < 0.001) and the heterogeneity (I^2^ = 76%, *p* < 0.001). Analysis of the remaining studies showed a trend toward publication bias, as indicated by the Begg’s (*p* = 0.04) and Egger’s (*p* = 0.16) test. Accordingly, the trim-and-fill method revealed six potential missing studies to be added to the left side of the funnel plot to obtain symmetry (Figure 5). The resulting SMD remained significant despite the further attenuation (SMD 0.73, 95% CI 0.46 to 0.99, *p* < 0.001).

**Figure 6:**
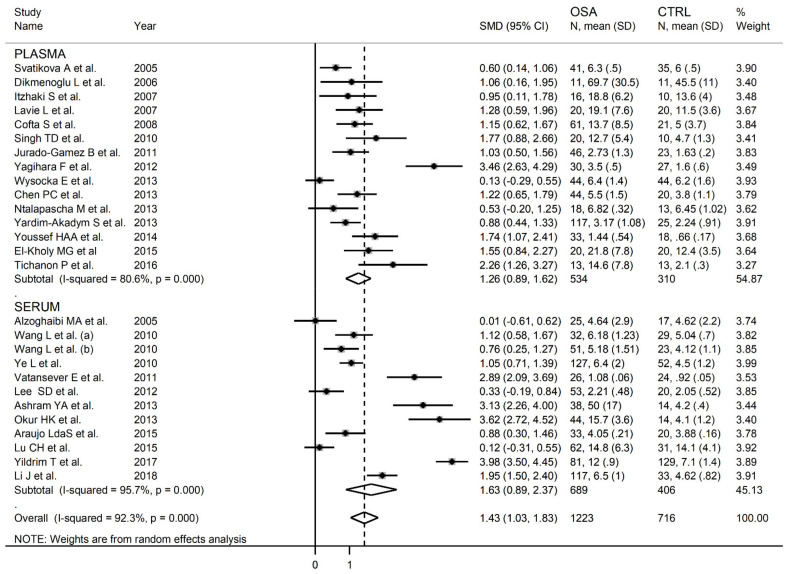
Forest plot of studies examining MDA concentrations in OSA and in controls according to the type of biological sample (plasma or serum).

**Figure 7:**
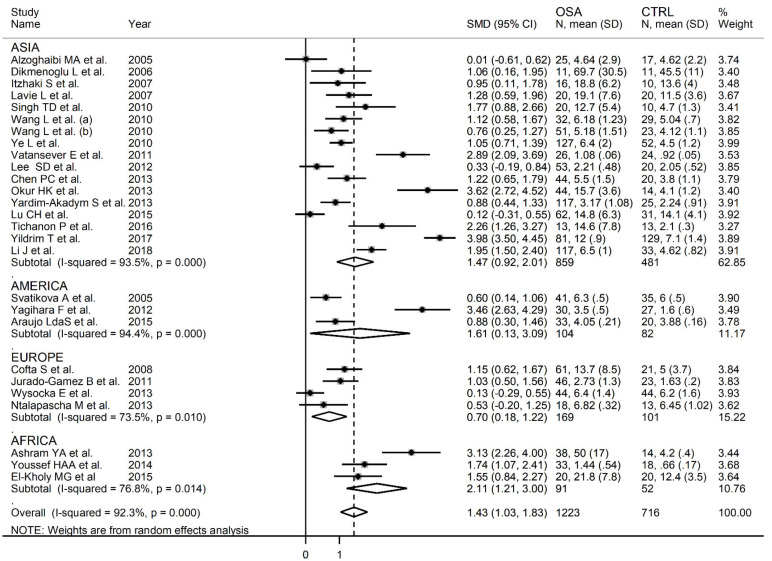
Forest plot of studies examining MDA concentrations in OSA and in controls according to the geographic area where the study was conducted.

In order to explore possible sources of heterogeneitywe investigated, by meta-regression analysis, the effects of different study characteristics including between-group difference in age, gender, body mass index (BMI), publication year, continent where the study was conducted (Europe, Africa, Asia and America), biological sample (plasma or serum), assay type used (spectrophotometric, high-performance liquid chromatography (HPLC) or enzyme-linked immunosorbent (ELISA)), low density lipoprotein (LDL), high density lipoprotein (HDL), total cholesterol (TC), triglyceride (TG) and glucose concentrations and measures of OSA severity (apnea-hypopnea index, AHI). Extreme heterogeneity was observed both in studies reporting serum (I^2^ = 95.7%, *p* < 0.001) and plasma (I^2^ = 80.6%, *p* < 0.001) concentrations, although the between-study variance was relatively lower in the latter (Figure 6). In meta-regression analysis, non-significant differences (t = 0.78, *p* = 0.44) in SMD values were observed between plasma (SMD 1.26, 95% CI 0.89 to 1.62, *p* < 0.001) and serum (SMD 1.63, 95% CI 0.89 to 2.37, *p* < 0.001) concentrations. Similarly, non-significant differences (t = 0.09, *p* = 0.93) in pooled SMD values were observed across Asian (SMD 1.47, 95% CI 0.92 to 2.01, *p* < 0.001), American (SMD 1.61, 95% CI 0.13 to 3.09, *p* < 0.001), European (SMD 0.70, 95% CI 0.18 to 1.22, *p* = 0.001) and African studies (SMD 2.11, 95% CI 1.21 to 3.00, *p* < 0.001) (Figure 7). However, a relatively lower heterogeneity was observed in African (I^2^ = 76.8%, *p* = 0.014) and European studies (I^2^ = 73.5%, *p* = 0.010). Gender (t = −0.10, *p* = 0.92), BMI (t = −0.41, *p* = 0.69), TC (t = −0.01, *p* = 0.99), LDL (t = −0.09, *p* = 0.93), HDL (t = 0.84, *p* = 0.42), TG (t = −0.71, *p* = 0.49) and glucose (t = 0.75, *p* = 0.47) were not associated with the SMD. Conversely, age (t = −2.06, *p* = 0.049), assay type (t = 2.31, *p* = 0.03) and publication year (t = 2.08, *p* = 0.048) were significantly associated with the SMD. 

**Figure 8:**
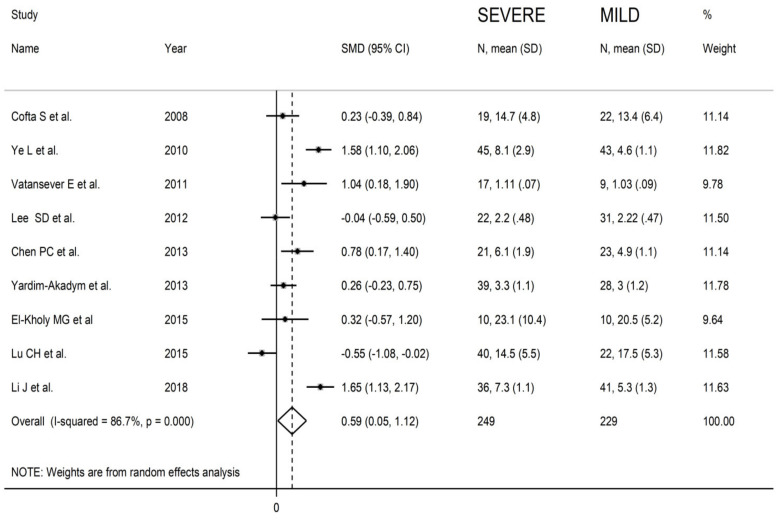
Forest plot of studies examining MDA concentrations in mild and severe OSA patients.

In order to evaluate the relationship between effect size and disease severity we performed a meta-analysis in a sub-group of nine studies reporting MDA concentrations in groups with different disease severity [32,35,37,38,42,45,47,49,52]. The forest plot for MDA concentrations in mild and severe OSA patients is reported in Figure 8. In two studies, patients with mild disease displayed significantly higher MDA values when compared to those with severe form (mean difference range, −0.04 to −0.55) [39,49]. By contrast, in the remaining seven studies, the MDA value was found to be higher in patients with severe disease (mean difference range 0.63 to 1.65), with a significant difference in four studies [35,37,42,52]. The pooled results showed that MDA concentrations were significantly higher in patients with severe disease (SMD 0.59; 95% CI 0.05 to 1.12, *p* = 0.03; I^2^ = 86.7%, *p* < 0.001) when compared with mild disease.

## 4. Discussion

Several studies have reported an increase in oxidative stress in OSA patients, both in terms of enhanced ROS production and as increased lipid peroxidation products [53,54,55,56]. Intermittent cycles of hypoxia and reoxygenation, which are the hallmarks of OSA, are likely to be involved in the intracellular generation of ROS that overwhelms the antioxidant defense system [53]. The consequent oxidation of various macromolecules provides further demonstration of the increased oxidative stress in OSA. Among them, lipids are mostly subject to oxidation [8]. Additionally, several authors have reported that continuous positive airway pressure therapy (nCPAP) not only reduces intermittent hypoxia but also attenuates lipid peroxidation in OSA patients [14,57]. A recent systematic review reported the presence of high concentrations of biomarkers of oxidative stress in OSA and a correlation between these biomarkers and the severity of the disease [58]. MDA is the most abundant aldehyde generated during the lipid peroxidation process and represents one of the most investigated markers of oxidative stress status in different lung diseases [12,59]. Thus, MDA may be useful for characterizing and monitoring the oxidative balance in OSA over time.

Our meta-analysis showed that pooled MDA concentrations were significantly higher in OSA patients compared to non-OSA controls. The observed SMD value, 1.43, suggests an effect size that is both biologically and clinically significant [60]. There was a large between-study heterogeneity, but, nevertheless, the sensitivity analysis showed that the overall SMD did not change significantly when each study was removed in turn. After excluding five studies that impaired the funnel plot symmetry the effect size was reduced but the SMD value remained significant and the magnitude of the heterogeneity decreased. In addition, the Begg’s test, but not the Egger’s test, revealed the presence of publication bias. Accordingly, the trim and fill analysis showed that six studies were potentially missing. Their addition to the left side of the funnel plot decreased the effect size without affecting the statistical significance. We conducted a subgroup analysis to further investigate the effect of different patient and study characteristics on the pooled SMD. Extreme heterogeneity was observed both in plasma and serum studies, whereas African and European studies were characterized by a relatively lower heterogeneity. Difference in age between controls and OSA; publication year; and assay type used were significantly related to the pooled SMD. In particular, the different assay type (spectrophotometric, ELISA or HPLC) may significantly account for between-study variance, with more specific assays as HPLC reporting larger between-group differences when compared with less specific spectrophotometric assays involving the use of thiobarbituric acid that is affected by interference with other chemical compounds in addition to MDA [61,62]. Several unreported factors might account for the observed heterogeneity including pre-analytical factors, e.g., time and conditions of sample storage; and specific methodologies and laboratory equipment for samples processing. Furthermore, control selection in case-control studies might have also affected the between-group difference. Whilst controls usually consisted of healthy subjects in some studies, their definition was not specified in others and they were simply described as “patients without OSA”. Additionally, the studies with unreported clinical parameters, e.g., smoking status, use of specific medications and presence of comorbidities that are known to affect oxidative/antioxidative balance and hence circulating MDA concentrations, might have accounted for the reported heterogeneity and publication bias. Unfortunately, the low number of articles reporting these data prevents further meta-regression analyses.

In the evaluation of the relationship between effect size and disease severity, the pooled SMD showed that MDA concentrations were significantly higher in patients with severe OSA compared to those with mild OSA. Although the number of studies that analyzed OSA patients according to the AHI is limited, our findings suggest the presence of a relation between severity of disease and oxidative stress.

The analysis of the effect of OSA treatment on circulating MDA concentrations, particularly in relation with different degrees of severity, represents an important issue. However, the lack of randomized controlled studies reporting this feature represents another limitation of our study. Nevertheless, our meta-analysis provides robust background information for the adequate design and conduct of further studies addressing this issue, particularly the usefulness of this marker for monitoring OSA and the effects of therapies. A recent meta-analysis has already demonstrated an increase of MDA in patients with OSA compared to the controls [63]. Even though the data obtained in terms of pooled SMD is similar in the two meta-analyses, our systematic search retrieved 26 studies involving a higher number of subjects compared to 14 studies analyzed by Fadaei R. et al. 2021 [63]. Additionally, the largest number of included articles permitted more factors relative to the investigation which might be related to the pooled SMD; these factors include publication year, geographic area, lipid profile and type of biological sample. Finally, our study also analyzed the concentrations of MDA in relation to disease severity. Therefore, our study provides a more complete and detailed picture with respect to MDA levels in OSA patients.

## 5. Conclusions

In conclusion, in our meta-analysis the significantly higher MDA concentrations observed in OSA patients, when compared to non-OSA subjects, supports the presence of lipid peroxidation in OSA. The significant heterogeneity observed warrants the use of standardized methods and adequate definitions of non-OSA subjects in future studies investigating the possible use of MDA as a clinical biomarker of OSA and to monitor the effects of interventions.

## Figures and Tables

**Table 1:** Participant characteristics of the studies included in the meta-analysis.

First Author Year. Country	Control								OSA							
	*n*	Age Mean ± SD Range	Gender (M/F)	BMI Mean	AHI%	ODI%	SaO2 Mean (%)/tSaO2 <90% (Min)	Comorbidities	*n*	Age Mean ± SD Range	Gender (M/F)	BMI Mean	AHI%	ODI%	SaO2 Mean (%)/tSaO2 <90% (Min)	Comorbities
Svatikova A et al. 2005. USA	35	47 ±2 -	35/0	26	NR	NR	- /1.1	No comorbidities	41	47 ±2 -	41/0	29.5	47	NR	- /13.2	No comorbidities
Alzoghaibi MA et al. 2005. Saudi Arabia	17	31 ±1.5 -	NR	23.4	NR	NR	NR	NR	25	50 ±2.2 -	NR	36.3	62	62.3	93 /-	NR
Dikmenoglu L et al. 2006. Turkey	11	46 - -	8/3	26.6	NR	NR	NR	DIAB 36.4% HTN 45.4%	11	50 - -	8/3	31.1	55	NR	NR	NR
Itzhaki S et al. 2007. Israel	10	50 ±4.4 -	8/2	28	7	0.9	NR	HTN /DIAB /DLP 37.5%	16	54 ±8.3 -	11/5	28	30	15	NR	HTN /DIAB /DLP 40%
Lavie L et al. 2007. Israel	20	42 ±10 -	16/4	26	6	NR	- /0.5	No comorbidities	20	42 11.1 -	16/4	26	29	NR	- /3.1	No comorbidities
Cofta S et al. 2008. Poland	21	52 ±7 -	11/10	33.4	NR	NR	NR	NR	61	53 ±6 -	43/18	32.5	23	NR	NR	NR
Singh TD et al. 2010. India	10	31 ±1.2 -	10/0	32.9	2	NR	NR	No comorbidities	20	44 ±2.4 -	20/0	24.5	61	NR	NR	HTN 15% DIAB 10%
Wang L et al. (a) 2010. China	29	69 ±4.2 -	27/2	26.8	3	NR	NR	HTN 13.8%	32	66 ±7.2 -	30/2	23.3	39	NR	NR	HTN 15.6%
Wang L et al. (b) 2010. China	23	45 ±12.3 -	20/3	25	3	NR	NR	HTN 13%	51	43 ±8.3 -	46/5	28.3	45	NR	NR	HTN 13.7 %
Ye L et al. 2010. China	52	45 0 -	37/15	26	2	3.5	NR	No comorbidities	127	45 ±11 -	102/25	26.3	36	38.7	NR	No comorbidities
Jurado-Gamez B et al. 2011. Spain	23	48 - 44-51	15/8	30	3	7	94 /-	DIAB 8%	46	45 - 40-47	34/12	31	46	49	93 /-	DIAB 4%
Vatansever E et al.2011. Turkey	24	47 ±8 -	24/0	28.4	2	NR	NR	No comorbidities	26	49 ±9 -	26/0	28.7	38	NR	NR	No comorbidities
Lee SD et al. 2010. South Korea	20	44 ±5.7 -	20/0	26.2	3	1.9	95.7 /0.04	HTN 4%	53	47 ±8.1 -	53/0	26.6	32	26	94.4 /9.2	No comorbidities
Yagihara F et al. 2012. Brazil	27	66 ±0.7 -	27/0	25.1	5	NR	94 /1.2	DLP 40.7 DIAB 26	30	66 ±0.7 -	30/0	27.9	38	NR	91 /76.7	DLP 40% DIAB 40%
Wysocka E et al. 2013. Poland	44	53 - 46-61	44/0	31.3	3	NR	NR	DIAB 50%	44	55 - 49-62	44/0	30	26	NR	NR	DIAB 50%
Ashram YA et al. 2013. Egypt	14	73 - 45-65	10/4	NR	NR	NR	NR	CVD 39.5% DIAB 42% HTN. 84.2%	38	75 - 33-87	22/16	NR	81	35	87 /23	No comorbidities
Chen PC et al. 2013. Taiwan	20	42 ±11 -	15/5	26	3.3	1	94 /-	No comorbidities	44	42 ±12 -	33/11	26.7	15	10	94 /-	No comorbidities
Ntalapascha M et al. 2013. Greece	13	50 ±13 -	13/0	28	3	8.61	93 /20	No comorbidities	18	49 ±10 -	18/0	31	58	61	90 /124	No comorbidities
Okur HK et al. 2013. Turkey	14	49 ±8.6 -	11/3	31.8	2.7	NR	90.4 /0	NR	44	44 ±13 -	40/4	30.5	37	47.2	76.6 /33	NR
Yardim-Akadym S et al. 2013. Turkey	25	43 ±8.2 -	14/11	27.2	3	28	94.4 /0.4	DIAB 11% HTN 23% DLP 48%	117	50 ±10.7 -	81/36	31.6	36	114	91 /54	DLP 32%
Youssef HAA et al. 2014. Egypt	18	45 ±12.7 -	4/14	42.8	2	8	94.4 /6	NR	33	52 ±11.5 -	23/10	44.3	19	41	89.6 /35.2	NR
El-Kholy MG et al 2015. Egypt	20	49 ±14.6 -	10/10	29.4	2	NR	- /3.3	No comorbidities	20	51 ±8.2 -	9/11	39	30	NR	- /28	No comorbidities
Araujo LdaS et al. 2015. Brazil	20	33 ±2 -	5/15	34.5	2.5	0.9	96.7 /0.06	No Comorbidities	33	40 ±1.5 -	20/13	34.4	20	13	95 /15.6	No Comorbidities
Lu CH et al. 2015 Taiwan	31	40 ±7.7 -	27/4	24.8	2.4	0.7	96.8 /-	No Comorbidities	62	42 ±10 -	54/8	25.5	41	31.5	94.8 /-	No Comorbidities
Tichanon P et al. 2016. Thailand	13	53 ±12.3 -	10/3	23.3	NR	NR	98.2 /-	HTN 69%	13	53 ±12.4 -	10/3	53	16	NR	94.2 /-	No Comorbidities
Yildrim T et al. 2017 Turkey	129	51 ±8.1 -	78/51	NR	NR	NR	NR	No Comorbidities	81	49 ±8.4 -	58/23	NR	34	NR	NR	No Comorbidities
Li J et al. 2018. China	33	42 ±10.1 -	29/4	25.8	4	3.6	91.8 /-	HTN 9%	117	45 ±10 -	105/12	25	25	28	94 /-	HTN 13%

BMI: body mass index (kg/m^2^); AHI: apnea-hypopnea index (events/h); ODI: oxygen desaturation index (events/h); SaO_2_: oxygen saturation; tSaO_2_ < 90% cumulative time during which the saturation of oxyhemoglobin was below 90%; CVD: cardiovascular diseases; HTN: hypertension; DIAB: diabetes; DLP: dyslipidemia; NR: not reported.

**Table 2:** MDA concentrations in the studies included in the meta-analysis.

First Author Year. Country	NOS (stars)	Matrix Type	Assay Type	MDA Mean (µmol/l) ± SD	
				Control	OSA
Svatikova A et al. 2005. USA	7	P	Sp	6.0 ± 0.5	6.3 ± 0.5
Alzoghaibi MA et al. 2005. Saudi Arabia	6	S	Sp	4.6 ± 2.2	4.6 ± 2.9
Dikmenoglu L et al. 2006. Turkey	8	P	HPLC	45.5 ± 11 ^#^	69.7 ± 30.7 ^#^
Itzhaki S et al. 2007. Israel	9	P	Sp	13.6 ± 4.0	18.8 ± 6.2
Lavie L et al. 2007. Israel	8	P	Sp	11.5 ± 3.6	19.1 ± 7.6
Cofta S et al. 2008. Poland	8	P	Sp	5.0 ± 3.7	13.7 ± 8.5
Singh TD et al. 2010. India	6	P	Sp	4.7 ± 1.3	12.7 ± 5.4
Wang L et al. (a) 2010. China	6	S	Sp	5.0 ± 0.7	6.2 ± 1.2
Wang L et al. (b) 2010. China	6	S	Sp	4.1 ± 1.1	5.2 ± 1.5
Ye L et al. 2010. China	8	S	Sp	4.5±1.2	6.4±2.0
Jurado-Gamez B et al. 2011. Spain	7	P	Sp	1.6±0.2	2.7±1.3
Vatansever E et al.2011. Turkey	7	S	HPLC	0.9 ± 0.05	1.1 ± 0.06
Lee SD et al. 2010. South Korea	7	S	Sp	2.1 ± 0.5	2.2 ± 0.5
Yagihara F et al. 2012. Brazil	9	P	Sp	1.6 ± 0.6	3.5 ± 0.5
Wysocka E et al. 2013. Poland	7	P	Sp	6.2±1.6	6.4±1.4
Ashram YA et al. 2013. Egypt	6	S	Sp	4.2 ± 0.4	50 ± 17
Chen PC et al. 2013. Taiwan	7	P	Sp	3.8 ± 1.1	5.5 ± 1.5
Ntalapascha M et al. 2013. Greece	7	P	Sp	6.5 ± 1.0	6.8 ± 0.3
Okur HK et al. 2013. Turkey	6	S	Sp	4.1 ± 1.2	15.7 ± 3.6
Yardim-Akadym S et al. 2013. Turkey	7	P	HPLC	2.2 ± 0.9	3.2 ± 1.1
Youssef HAA et al. 2014. Egypt	7	P	Sp	0.66 ± 0.17	1.44 ± 0.54
El-Kholy MG et al 2015. Egypt	7	P	Sp	12.4 ± 3.5	21.8 ± 7.8
Araujo LdaS et al. 2015. Brazil	7	S	Sp	3.88 ± 0.16 ^§^	4.05 ± 0.21 ^§^
Lu CH et al. 2015 Taiwan	8	S	Sp	14.1 ± 4.1	14.8 ± 6.3
Tichanon P et al. 2016. Thailand	8	P	Sp	2.1 ± 0.3	14.6 ± 7.8
Yildrim T et al. 2017 Turkey	6	S	ELISA	7.1 ± 1.4	12 ± 0.9
Li J et al. 2018. China	7	S	Sp	4.6 ± 0.8	6.5 ± 1.0

NOS: Newcastle-Ottawa quality assessment scale for case-control studies; P: plasma; S: serum; Sp: spectrophotometric; ELISA: enzyme-linked immunosorbent assay; HPLC: high performance liquid chromatography; NR: not reported; ^#^ nmol/L; ^§^ ng/mL.

## Supplementary Materials

The following are available online at https://www.mdpi.com/article/10.3390/antiox10071053/s1, Figure S1: Bar chart showing MDA concentrations means in OSA patients and controls in the studies included in the meta-analysis. The assay type used is indicated above the bars. Sp: spectrophotometric. ELISA: enzyme-linked immunosorbent assay; HPLC: High Performance Liquid Chromatography. ***** Statistical significance in presence of *p* < 0.05; # nmol/L; ^§^ ng/mL.
