# Supplementary material for: Circulating Malondialdehyde Concentrations in Obstructive Sleep Apnea (OSA): A Systematic Review and Meta-Analysis with Meta-Regression
Source: Antioxidants (Basel). 2021 Jun 29;10(7):1053. doi: 10.3390/antiox10071053 (PMC8300833; doi:10.3390/antiox10071053)
Supplement: Supplementary file 1 [file antioxidants-10-01053-s001.zip › antioxidants-1218857-supplementary.pdf]

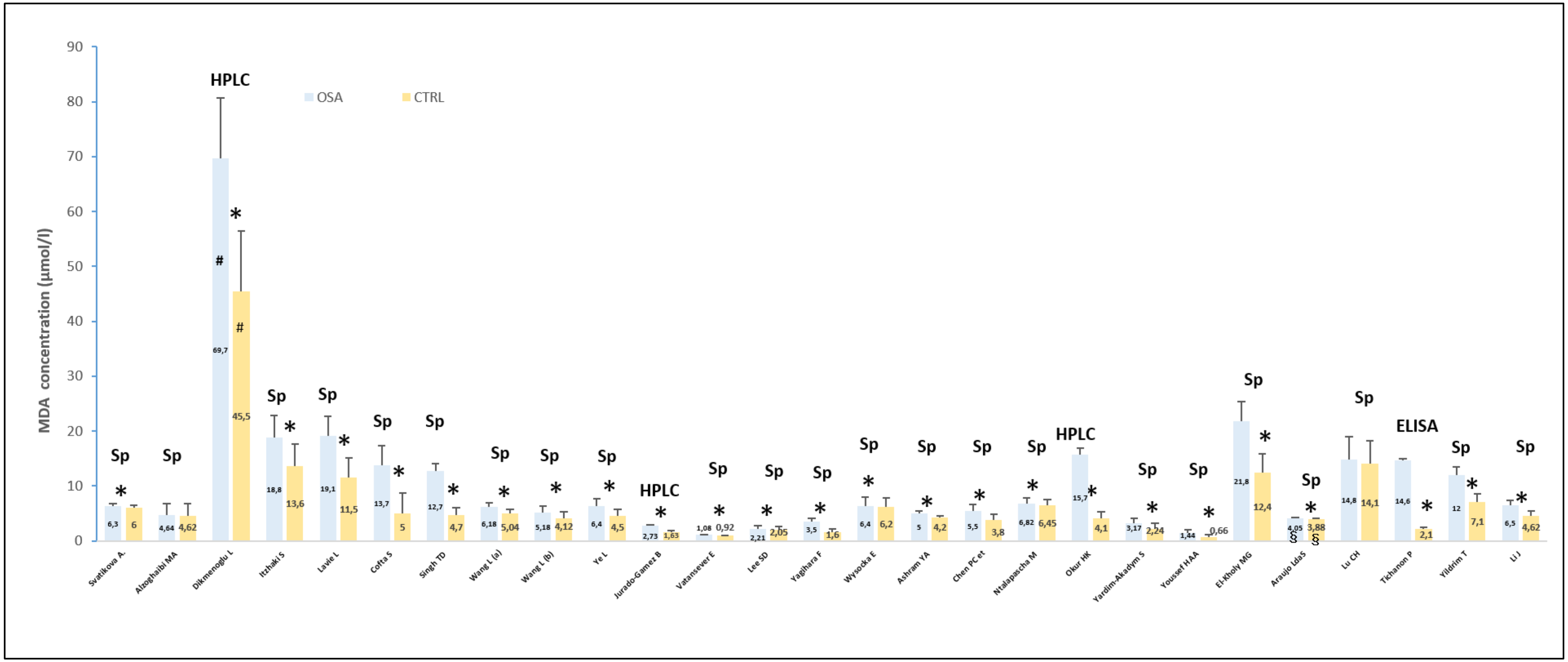

Supplementary Figure S1**.** Bar chart showing MDA concentrations means in OSA patients and controls in the studies included in the meta-analysis. The assay type used is indicated above the bars.

Sp: spectrophotometric. ELISA: enzyme-linked immunosorbent assay; HPLC: High Performance Liquid Chromatography.

* Statistical significance in presence of $p < 0.05$;

\# nmol/l;

§ng/ml.
